# An Orthodontic Approach for Garre’s Sclerosing Osteomyelitis of the Mandible

**DOI:** 10.3390/ijerph18063159

**Published:** 2021-03-18

**Authors:** Ioan Barbur, Simion Bran, Mihaela Baciut, Gabriel Armencea, Alexandra Iulia Aghiorghiesei, Tudor-Sergiu Suciu, Adina Maria Barbur, Horia Opris, Grigore Baciut, Cristian Dinu

**Affiliations:** 1 Department Maxillofacial Surgery and Implantology, Iuliu Hatieganu University of Medicine and Pharmacy, 37 Cardinal Iuliu Hossu Street, 400029 Cluj-Napoca, Romania; drbarbur@gmail.com (I.B.); mbaciut@yahoo.com (M.B.); garmencea@gmail.com (G.A.); suciu_ts@yahoo.com (T.-S.S.); gbaciut@umfcluj.ro (G.B.); dinu_christian@yahoo.com (C.D.); 2 Department of Prosthodontics and Dental Materials, Iuliu Hațieganu University of Medicine and Pharmacy, 32 Clinicilor Street, 400006 Cluj-Napoca, Romania; ada_irimie@yahoo.com; 3 Ortolife Orthodontic Private Practice, 8 Andrei Muresanu Street, 400071 Cluj-Napoca, Romania; drbarburadina@yahoo.com

**Keywords:** osteomyelitis, orthodontics, functional therapy, mixed dentition

## Abstract

The nonsuppurative osteomyelitis of the mandible is a rare condition that can occur in children due to low-grade inflammatory processes, dental cavities, periodontal lesions as well as the eruption process of the teeth. We submit a case report involving the orthodontic management of a 9-year-old female patient who presented in our service in the mixed dentition period with diagnosed Garre’s sclerosing osteomyelitis of the entire mandibular body. After a full work-up, the following symptoms and signs were noted: bilateral temporomandibular joint (TMJ) pain, loss of the leeway space, anterior open bite, distalization of the secondary maxillary right canine, nail biting and tongue thrust. Our orthodontic objectives were to relieve the TMJ pain, limit the eruption process of the teeth and to diminish the evolution of the osteomyelitis, reduce the growth of the inferior lower third of the face and to prevent further invasive treatment of the patient. In the first phase of treatment, we established a centric relationship using an orthopedic appliance (occlusal splint) and physiotherapy to deprogram the muscles and the TMJ. Throughout the second phase of treatment, we used orthopedic appliances to inhibit the overeruption of the secondary molars. After another year of treatment, the osteomyelitis lesions were under control with the permanent teeth in final position, good facial esthetic and as a functional result, no root resorption. We can conclude that by using low physiological forces to direct and control the growth pattern, good results could be obtained in stabilizing and controlling the sclerosing osteomyelitis of the mandible.

## 1. Introduction

Osteomyelitis is defined as the infection and inflammation of the bone structures due to bacteremia, trauma, surgery or surrounding soft tissue infection with a wide variety of presenting signs and symptoms [1,2]. Diagnosis is based on the clinical presentation, history of predisposing factors, imaging studies and microbiologic tests [3]. Bone biopsy is usually required to confirm the investigations [4]. Treatment usually consists of antibiotic therapy with or without surgical debridement of the necrotic tissue [5,6].

Garre’s osteomyelitis can be found in the literature with different entities, nonsuppurative ossifying periostitis or periostitis ossificans. It is an uncommon disease occurring in children or young adults due to their increased vascularity and regenerative capabilities and is considered a chronic sclerosing osteomyelitis with a concomitant persistent periosteal inflammatory reaction [7]. Some consider it as a form of chronic recurrent multifocal osteomyelitis (CRMO). It can occur in the tibia and in the maxillofacial skeleton. In the mandible, the most common cause is low-grade inflammatory processes such as erupting molars, carious teeth, infection of the molars, pericoronal infections or infections following teeth extractions. Initial presentation of such patients typically includes a bony hard swelling of the posterior mandible with the soft tissues surrounding the affected area appearing normal, without purulence, drainage or erythema [8]. The radiography outlines paracortical bone formation with multiple lytic lesions associated with sclerosis (“onion skin”), which are suggestive of but are not pathognomonic. Diagnosis can be presumed radiologically, and the biopsy confirms the features of chronic osteomyelitis with negative bacterial cultures [9]. Treatment of Garre’s osteomyelitis includes the removal of the etiologic agent, antibiotic treatment and debridement. The clinical course usually presents as sudden onset with progression, spontaneous regression and remodeling [9]. Differential diagnosis of Garre’s osteomyelitis includes cortical hyperostosis (Caffey disease), osteosarcoma and Ewing sarcoma [10].

The deciduous teeth have an important role in guiding the eruption of the permanent teeth and their premature loss can have severe effects on developing the occlusion with teeth crowding, impaction, ectopic eruption, over-eruption of unopposed teeth and center line discrepancies [11].

The research conducted in all major medical databases (PubMed, Scopus, Web of Science) for this article revealed no publications regarding the orthodontic management of cases involving Garre’s osteomyelitis.

This article presents and extensively debates on the management of a patient diagnosed with Garre’s osteomyelitis in the mixed dentition period and its orthodontic therapy.

## 2. Case Presentation

Approval was obtained from the Ethical Committees of the University of Medicine and Pharmacy “Iuliu Hatieganu” Cluj-Napoca (345/16 November 2020) and informed consent from the patient and her mother were taken for the publication of this case report.

### 2.1. Initial Diagnosis and Initial Surgical Management

A female patient aged 9 (born 1995) was referred to the Maxillofacial Department in 2014 because of rapid onset of pain, swelling of the jaw and difficulty with eating. Clinical examination revealed no pus, abscess or fistulae. Further investigation reported a severe enlargement of the body, ramus and mandibular arch and associated pain. No sensitivity alteration was noted at the inferior alveolar nerve innervation territory.

**Figure 1:**
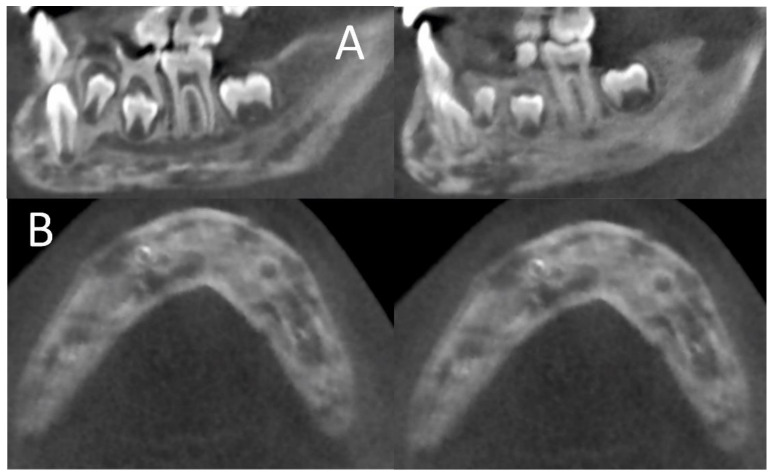
Initial Cone beam computed tomography (CBCT) examination shows osteolytic and sclerosis lesions (“onion skin”); A—sagittal sections through the body and ramus of the mandible; B—cross sections through the body of the mandible.

**Figure 2:**
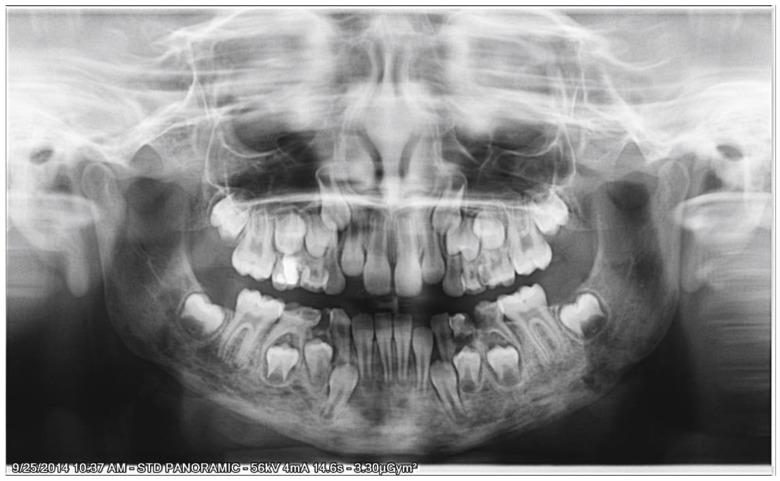
Panoramic radiograph showing the “onion-skin” appearance of the bone at full length of the mandible.

Upon examining the cone beam computer tomograph (CBCT) (Figure 1) and panoramic X-ray (Figure 2), multiple poorly differentiated radiolucencies with diffuse radiopacities were observed spanning the entire length of the mandible body from one angle to the other. A sclerotic or sclerotic-permeative pattern, as well as a periosteal reaction, was also seen. The lesions did not seem to affect the teeth and the mandibular canal.

**Figure 3:**
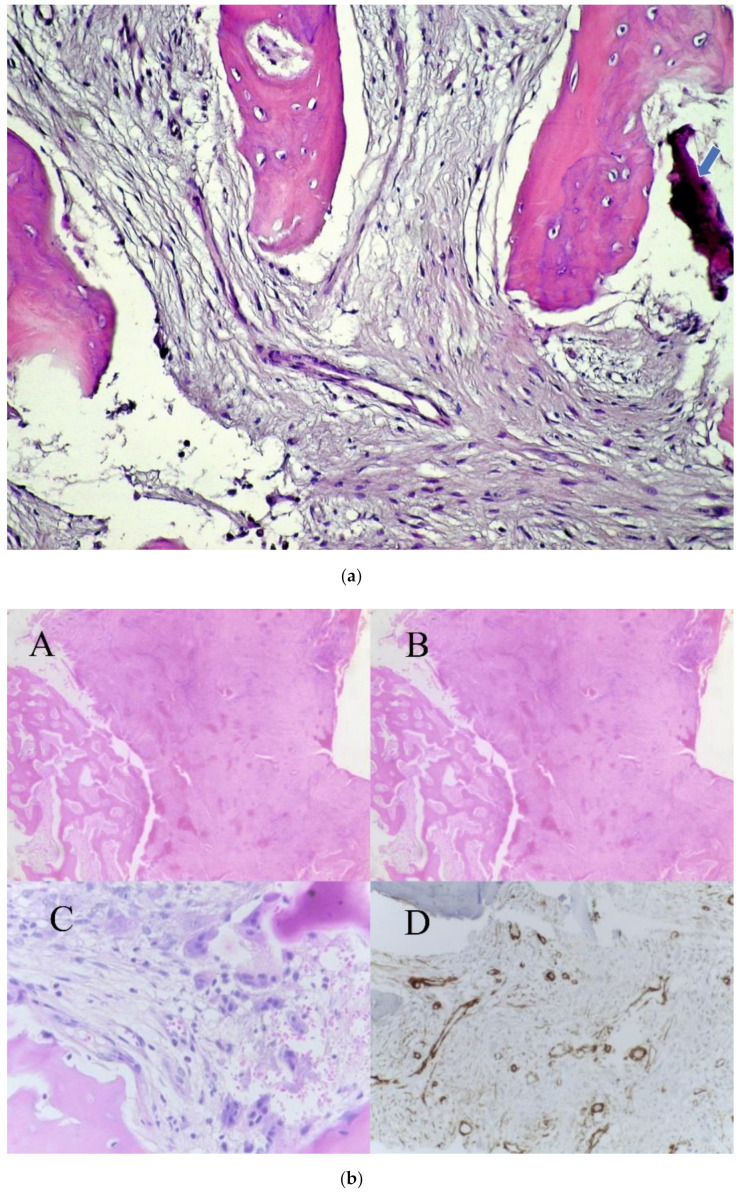
(**a**)—Trichrome Masson staining: bone trabeculae with medullar fibrosed tissue (arrow show a fragment of bone sequestrum). (**b**)—Biopsy Hematoxylin-eosin staining: eosinic deposits at 40× magnification (A), at 100× eosinic deposits and (B) 400× with giant cells (C); Alpha smooth muscle actin (α-SMA) staining showing fusiform cells (D).

As a first empirical treatment, large doses of wide spectrum antibiotics were used, and bone biopsy was taken from the mandible. The Trichrome Masson, hematoxylin-eosin and the immunohistochemistry staining revealed chronic sclerosing osteomyelitis (Figure 3a,b).

Although no suppurative process was clinically evident, microbiological samples were collected, with no conclusive result. Under wide spectrum antibiotic therapy slowly favorable evolution was noted, but the thickening of the periosteum was still present with the relapse of the symptoms several weeks after the treatment ended.

In 2016, local surgical implantation of gentamicin-PMMA (Polymethyl methacrylate) beads (Septopal; Merck GmbH, Darmstadt, Germany; Biomet GmbH, Berlin, Germany) to the right and left body of the mandible was decided in an attempt to stop the evolving osteomyelitis.

### 2.2. Orthodontic Treatment

#### 2.2.1. Initial Examination

Due to loss of several deciduous teeth in the lateral region and pain reported by the patient in the temporomandibular joints (TMJs) bilaterally, the patient was referred for orthodontic assessment. Digital photographs and dental impressions were taken at her first visit and, moreover, the bite was registered in centric relation. In addition, the patient received indications for radiographs, CBCT and Magnetic resonance imaging (MRI) for the TMJs.

**Figure 4:**
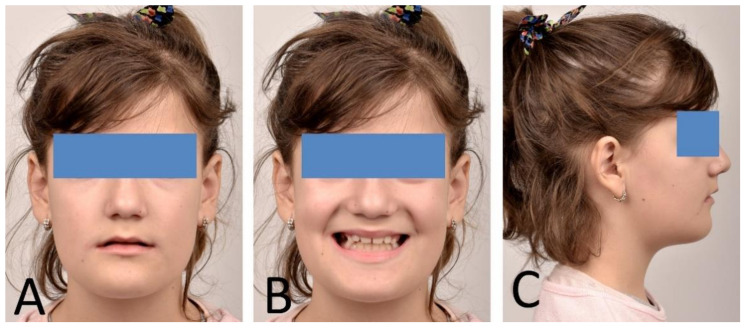
Pre-treatment extra-oral photographs; (**A**)—Frontal view; (**B**)—Smile; (**C**)—lateral view.

The initial extraoral facial examination revealed an increased lower face height and a straight profile, reduced static and dynamic upper frontal teeth exposure, lip incompetence (3 mm). The midline of the mandible and maxilla corresponded with the midline of the face (Figure 4).

Intraoral examination revealed partial destruction on the lateral deciduous supporting areas resulting in a loss of the leeway space bilaterally and bimaxillary shortening of the dental arches, accentuated Spee curve and poor oral hygiene.

**Figure 5:**
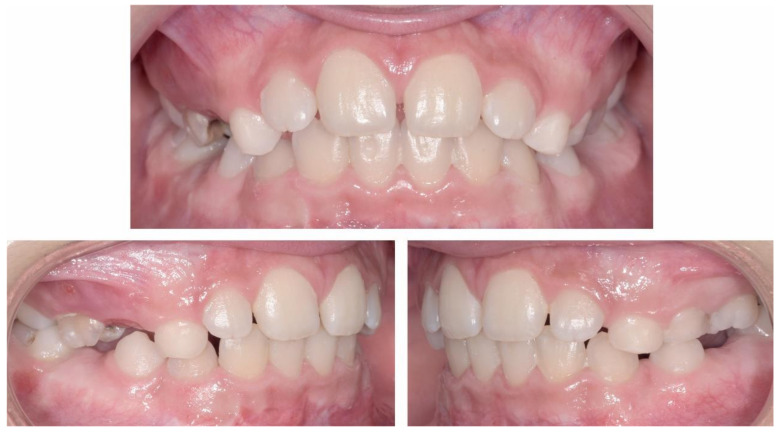
Pre-treatment intra-oral photographs.

**Figure 6:**
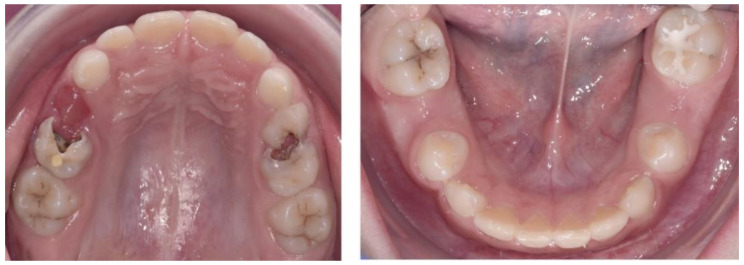
Pre-treatment intra-oral occlusal photographs.

The occlusal exam found a half a cusp distalization of the secondary right mandibular canine, due to the leeway space loss, a class I relationship on the left canine and a molar class I relationship on both sides (Figure 5 and Figure 6). Tongue thrust and a habit of nail biting (onychophagia) were also noted at the functional exam.

The dental exam also revealed extensive carious lesions in the temporary maxillary second right molar and on both temporary left maxillary molars. The secondary inferior first molar had an incipient carious lesion on the occlusal surface (the tooth is vital for the vitality test).

The initial cephalometric analysis revealed a decreased articular angle, increased gonial-jaw angle and reduced corpus length, which signify a normal growth pattern. The anteroposterior relationship of the mandible and the maxilla was normal.

**Figure 7:**
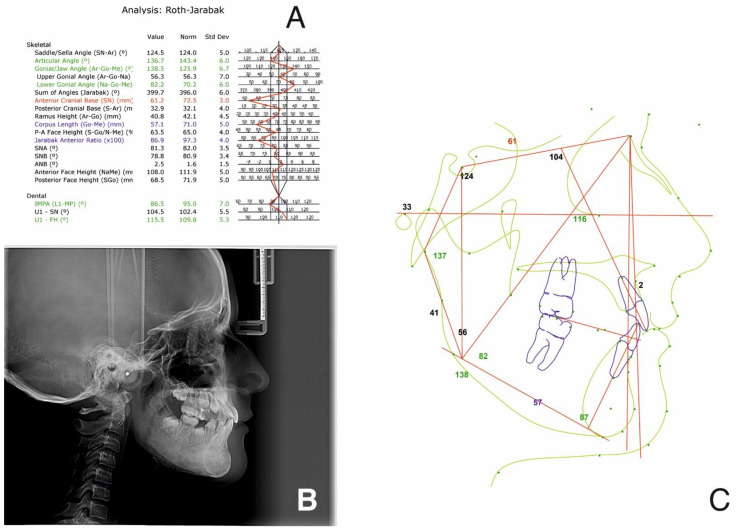
Pre-treatment cephalometric analysis: Roth Jarabak cephalometric analysis (**A**), cephalometric radiograph (**B**), landmarks and reference lines of the cephalometric analysis (**C**).

From a dental point of view, the lower incisors were retrusive, due to the premature loss of deciduous teeth. The following were also noted: a tendency of inversion of the normal lordosis of the vertebrae and posterior rotation of the cranium (Figure 7).

**Figure 8:**
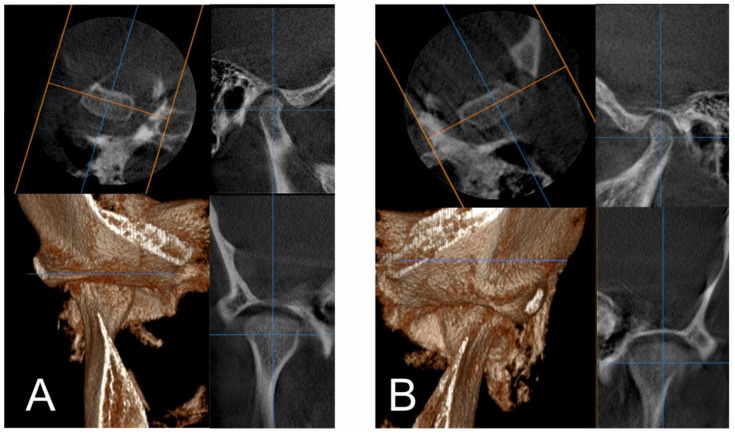
Pre-treatment TMJ aspect on CBCT: right (**A**) and left (**B**) TMJ with coronal, sagittal, axial cross-sections and 3d reconstruction.

**Figure 9:**
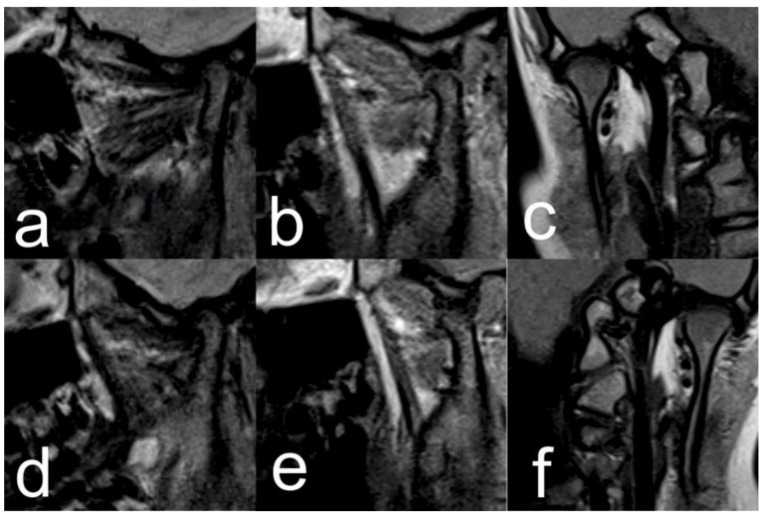
Pre-treatment TMJ MRI: Right TMJ sagittal view closed mouth (**a**), open mouth (**b**) and closed mouth coronal view (**c**); Left TMJ sagittal view closed mouth (**d**), open mouth (**e**), closed mouth coronal view (**f**).

CBCT and MRI analyses showed atrophic disks bilaterally and perforation on the right disk with antero-lateral displacement. On the left TMJ, an anterior-lateral displacement was noted. Bilateral condylar resorption and active erosions, without any inflammatory signs, were also seen (Figure 8 and Figure 9).

Cast models were mounted in centric relation du jour using a face bow and articulator. A premature contact at 1.6–4.6 and the lateral shift of the mandible were noted. This reveals a Centric Occlusion—Centric Relation discrepancy, which might have determined the TMJ symptomatology. Upon analysis, an overjet of 1–2 mm and an overbite of 1–2 mm was found with an accentuated Curve of Spee.

One of the causes of the premature contact might be the premature loss of the deciduous teeth (1st and 4th quadrant), with a consecutive migration of the adjacent teeth.

#### 2.2.2. Treatment Objectives

1. Relieve the TMJ pain, stabilize the mandible and muscle relaxation (physiotherapy and splint therapy);
2. Orthopedic treatment to ensure a normal growth and development of the maxilla and the mandible, and the correct eruption of the permanent teeth (class I for canine and molar);
3. Diminishing the growth of the inferior lower third of the face;
4. Limiting the evolution of the osteomyelitis process by preventing further invasive treatment of the patient (braces, skeletal anchorage, any other forces that are beyond physiological which might increase the osteomyelitis process);
5. Deconditioning of the tongue trust and nail biting and improving oral hygiene.

#### 2.2.3. Treatment Planning and Treatment Alternatives

Care was taken to avoid the usage of a fixed appliance after the mixed dentition phase, or any skeletal anchorage. To this end, the treatment had to use appliances to direct the growth pattern, in order to utilize only low physiologic forces (biting and tongue forces). These were intended to slowly close the open bite, to autorotate the mandible, to obtain a class I occlusion for the molars and canines bilaterally. Caution was also taken to prevent any potential resorption of the roots. All this was done to avoid any osteo-periosteal reaction and the possibility of aggravating the disease. Another very important objective, while planning the orthodontic treatment, was to adjourn unnecessary surgery including teeth extractions or wisdom teeth removal. Special consideration was taken not to use an unphysiological high pull force from a head-gear device to intrude the lateral teeth to close the open bite.

Usually, in cases with loss of leeway space, an alternative would be to regain lost space with the help of fixed orthodontic appliances (braces) and space opening mechanics, but this treatment alternative was not advisable for the patient since it might produce exacerbation of the osteomyelitis.

The prognosis of this disease is unfavorable with severe pain of the jaw, TMJ discomfort and pain, difficulty with eating and with hygiene. The osteomyelitis will accentuate and fistulae can appear. Orthodontic-wise, due to the leeway space loss, crowding will appear in the permanent dentition with the development of open-bite and a hyperdivergent growth pattern. The orthodontic treatment, then, will have to include teeth extractions, which are a risk factor of aggravating the osteomyelitis. Additionally, extensive fixed appliances will have to be used alongside skeletal anchorage. All this determines the accentuation of the symptomatology of the underlying disease.

### 2.3. Treatment Progress

#### 2.3.1. First Treatment Phase—2016—TMJ Splint Therapy

**Figure 10:**
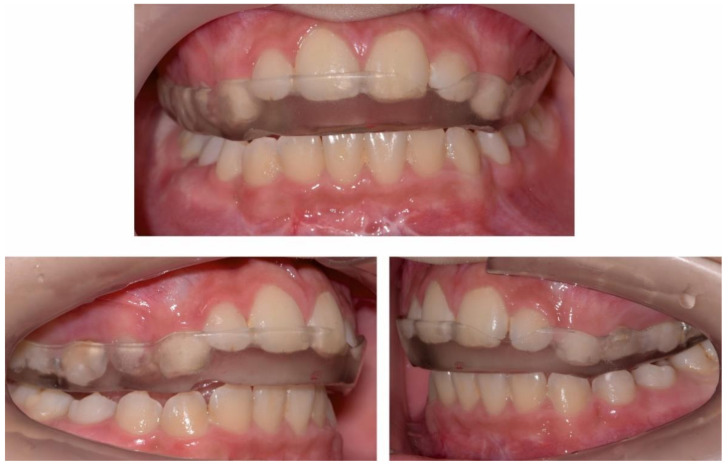
Maxillary one-piece acrylic splint.

**Figure 11:**
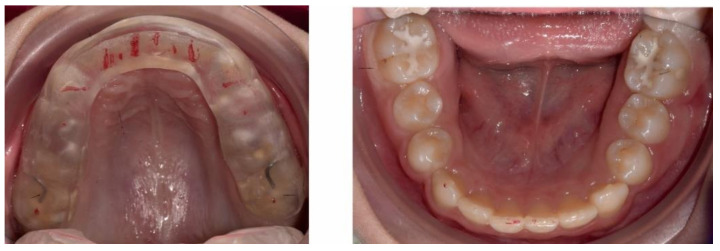
Intra-oral occlusal photographs with maxillary splint.

Due to the symptomatic TMJs and the modifications revealed on the MRI and CBCT, treatment began with full-time wearing of an acrylic splint and physiotherapy, to deprogram the muscles and the TMJs and to establish the centric relation. A full coverage maxillary splint divided into three sections (one frontal and two lateral) was manufactured according to a centric bite registration (Figure 10 and Figure 11).

**Figure 12:**
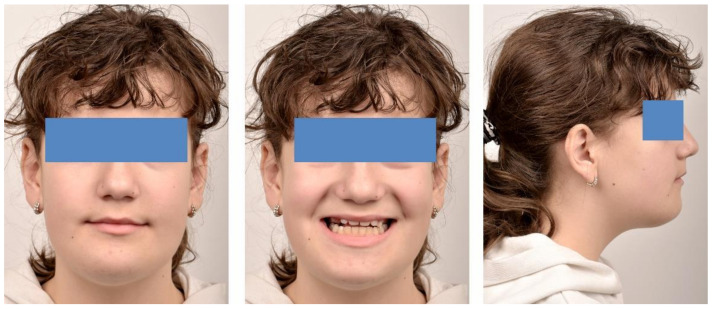
Post-splint therapy evaluation—extra-oral photographs.

**Figure 13:**
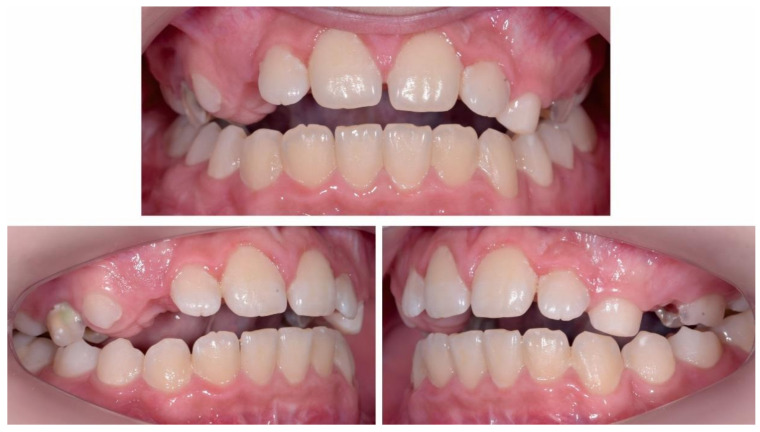
Post-splint therapy evaluation—intra-oral photographs.

**Figure 14:**
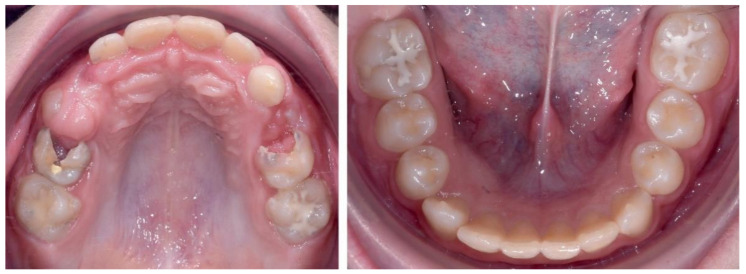
Post-splint therapy evaluation—intra-oral occlusal photographs.

**Figure 15:**
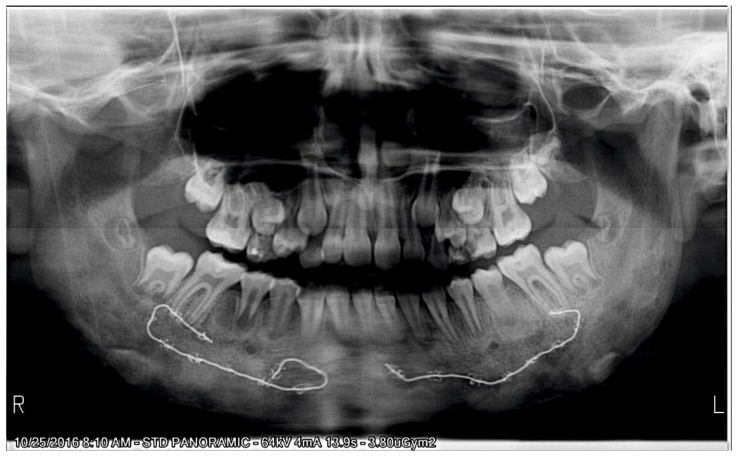
Post-splint therapy evaluation—panoramic radiograph.

After 13 months of physiotherapy and splint therapy (Figure 12, Figure 13, Figure 14 and Figure 15), centric relation was established with a premature contact on teeth 2.6–3.6 and an anterior open bite of 3–5 mm, a lateral open bite of 3 mm and still a small lateral shift of the mandible to the right. As seen on the panoramic X-ray (Figure 15) the gentamicin-PMMA beads were applied in the left and right body of the mandible.

**Figure 16:**
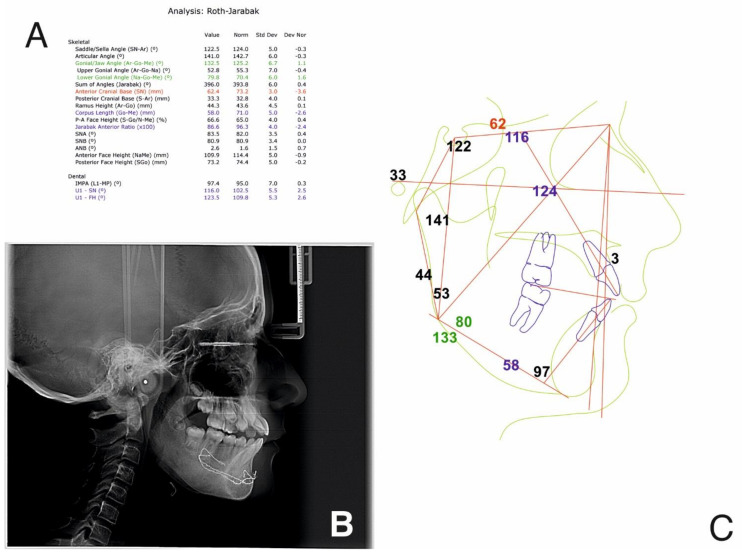
Post-splint therapy evaluation—cephalometric analysis: Roth Jarabak cephalometric analysis (**A**), cephalometric radiograph (**B**), landmarks and reference lines of the cephalometric analysis (**C**).

The cephalometric analysis (Figure 16) revealed an articular angle in the normal range, while all the other parameters remaining the same. Additionally, the natural curvature of the cervical spine was re-established and the space between C0, C1 and C2 was restored.

#### 2.3.2. Second Phase of Treatment—2017—Bite-Blocks and Transpalatal Arch

**Figure 17:**
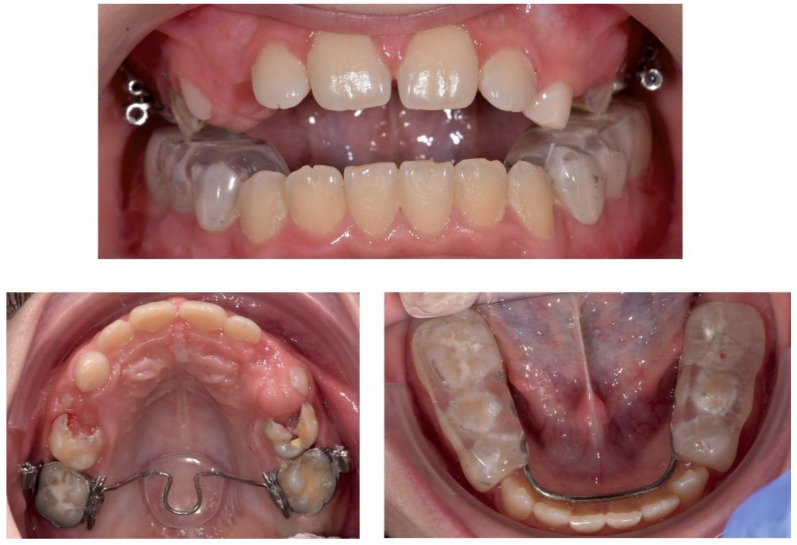
Transpalatal arch with acrylic button and mandibular acrylic splint.

**Figure 18:**
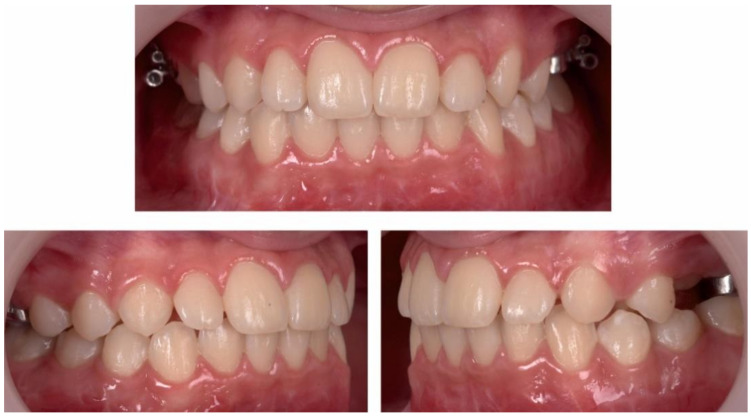
Intra-oral aspect after 15 months of treatment with TPA and mandibular acrylic splint.

**Figure 19:**
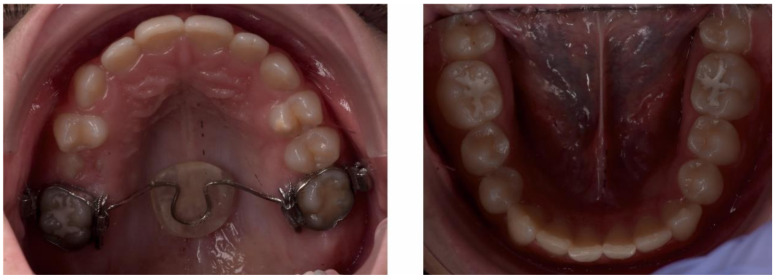
Intra-oral aspect after 15 months of treatment with TPA and mandibular acrylic splint—occlusal photographs.

A mandibular removable arch with bite-blocks (worn 24/7, even when eating) manufactured in centric relationship was used to inhibit the overeruption of the lateral mandibular teeth and a transpalatal arch (TPA) with an acrylic button on the permanent upper first molars was used to intrude the permanent upper molars by means of the physiological forces of the tongue when swallowing (Figure 17, Figure 18 and Figure 19).

Due to good local response and decreasing osteoclastic lesions, the gentamicin-PMMA beads (Septopal) were surgically removed in 2017. The biteblocks were used until the permanent dentition fully erupted.

## 3. Results

**Figure 20:**
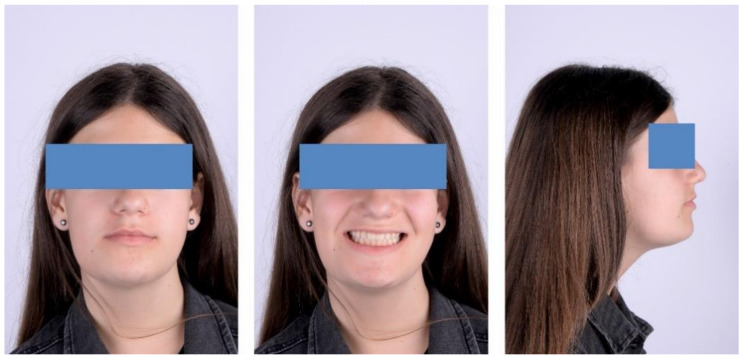
Extra-oral aspect at the end of treatment.

**Figure 21:**
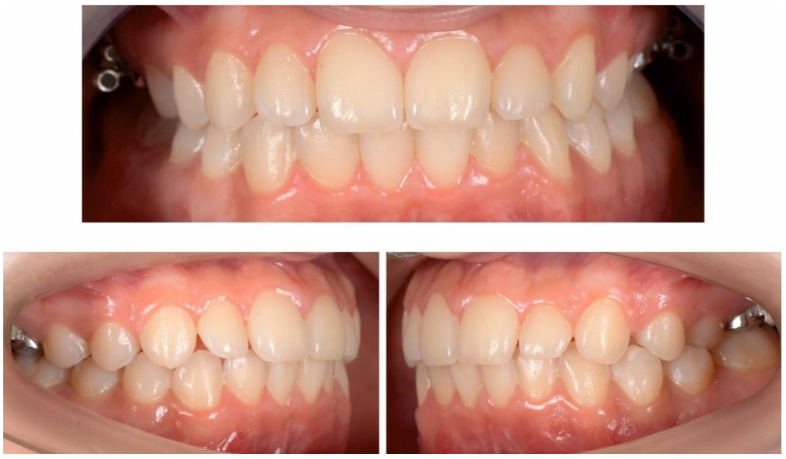
Intra-oral aspect at the end of treatment.

**Figure 22:**
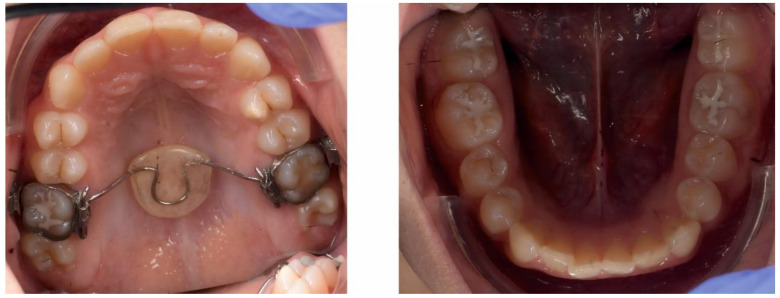
Intra-oral occlusal aspect at the end of treatment.

After a year of treatment, the patient displayed an esthetically pleasing smile and improved facial esthetics (Figure 20). Intraoral examination of the occlusion revealed a class I molar and canine relationship on both sides, and adequate overjet and overbite, with a flattened curve of Spee (Figure 21 and Figure 22).

**Figure 23:**
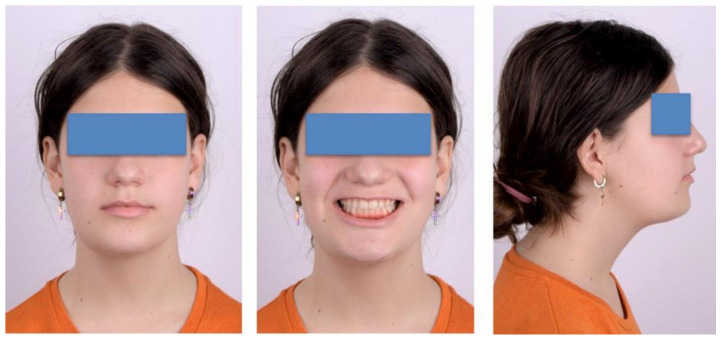
Extra-oral aspect at the one-year follow-up appointment.

At the one-year follow-up appointment, in 2019, the clinical exam revealed an improvement in facial esthetics, due to the reduction of the mandible. The profile remained straight, and smile esthetics were pleasing (Figure 23).

**Figure 24:**
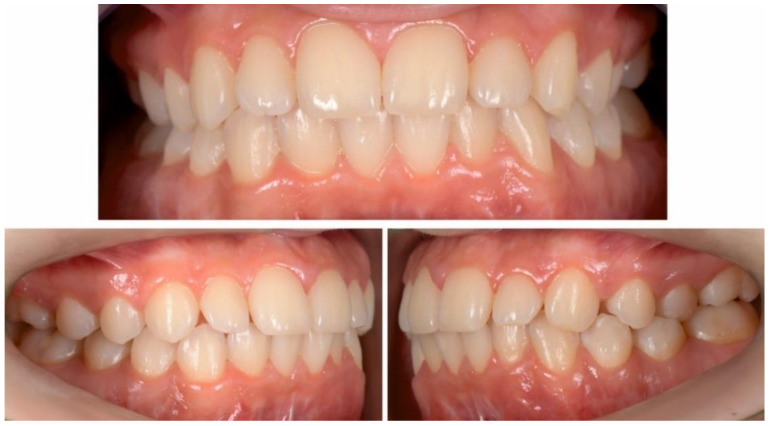
Intra-oral aspect at the one-year follow-up appointment.

**Figure 25:**
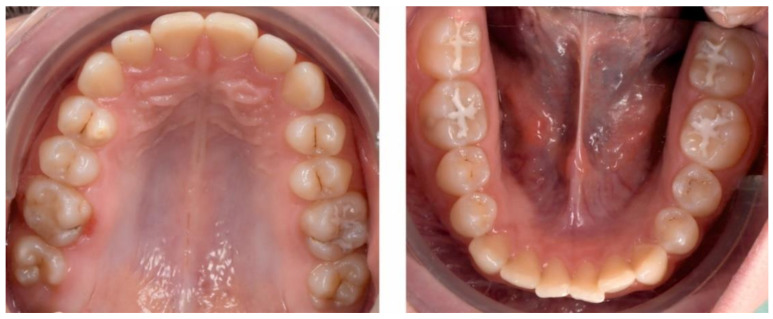
Intra-oral occlusal aspect at the one-year follow-up appointment.

The intraoral examination showed a class I canine and molar occlusal relationship on both sides, with good oral hygiene, normal swallowing and no nail biting, so the treatment results remained stable (Figure 24 and Figure 25).

**Figure 26:**
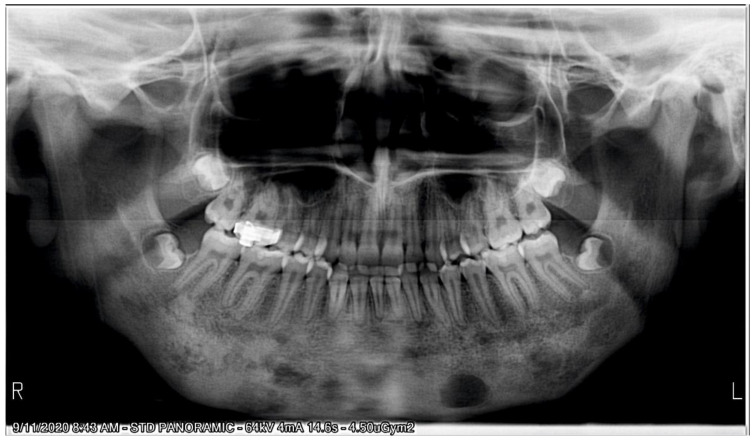
Panoramic radiograph at the one-year follow-up appointment.

The final panoramic radiograph shows no root resorption and roots are parallel, with only few osteoclastic lesions are present, and condensation of the entire length of the mandible is visible (Figure 26).

**Figure 27:**
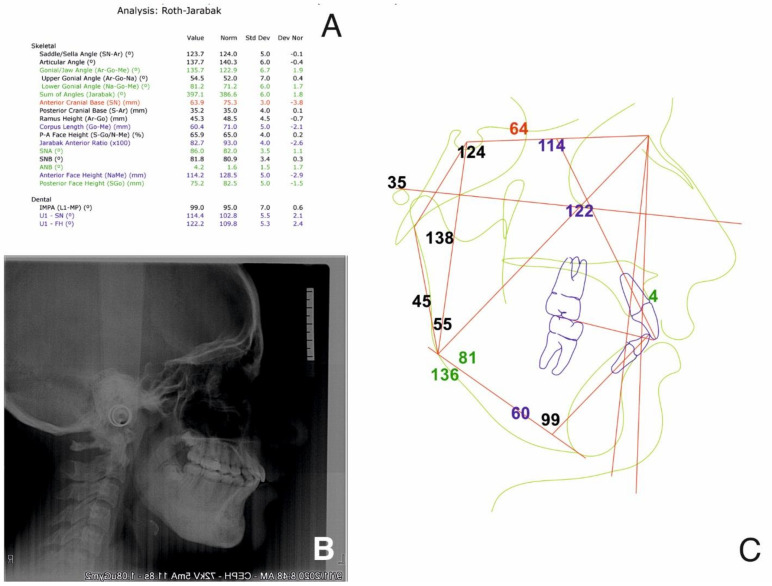
Cephalometric analysis at the one-year follow-up appointment: Roth Jarabak cephalometric analysis (**A**), cephalometric radiograph (**B**), landmarks and reference lines of the cephalometric analysis (**C**).

Cephalometric analysis at the end of treatment shows that the gonial-jaw angle has remained constant, while the articular angle is now in the normal range. Corpus length is still short, but has slightly increased since the beginning of treatment, meaning that there is a normal anterior growth pattern. From the dental point of view, lower incisors position is now normal, and there is protrusion of the upper incisors, due to the autorotation of the mandible and protrusion of the lower incisors (Figure 27).

**Figure 28:**
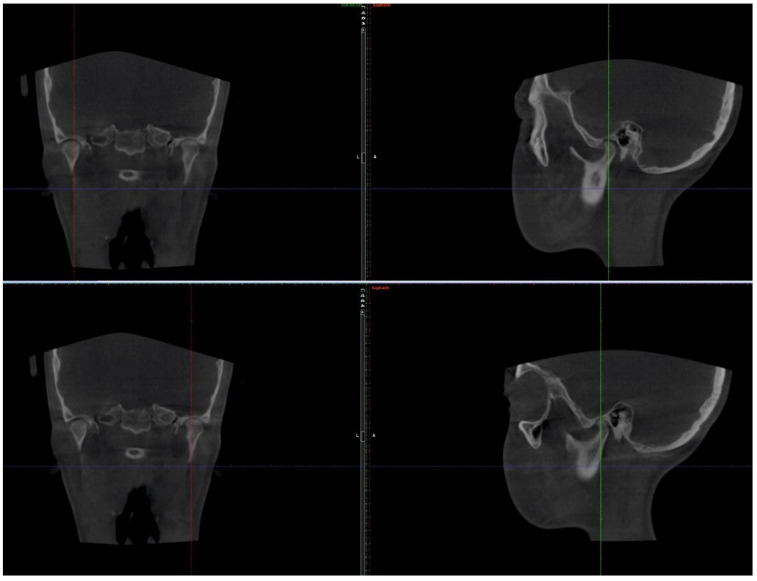
TMJ aspect on CBCT at the one-year follow-up appointment.

Stability was also noted for the TMJs (Figure 28), with no further pain or discomfort.

## 4. Discussion

Garre’s osteomyelitis can occur due to mild irritation or infection in the mandibular area [12]; therefore, in order to avoid acceleration of the disease, the main goals of the orthodontic treatment for this patient were to avoid any osteo-periosteal reaction. To do so, the least invasive treatment was selected and performed, with the purpose of maintaining a good natural growth pattern and guiding tooth eruption, while avoiding further orthodontic treatments with fixed appliances and skeletal anchorage. Infections after tooth extractions and pericoronitis of third molars have also been linked to this affection [13], so tooth extractions were also avoided.

Frequently used for treating Garre’s osteomyelitis, root canal treatment is cited by numerous authors as a direct link to this specific pathology, with a good and stable result, which confirms the etiology [14,15,16,17].

The etiology of Garre’s osteomyelitis is widely disputed; a study by Groot et al. [18] researched the masseter inhibitory reflex of such patients, which supported the idea that this pathology is in fact a chronic tendoperiostisis caused by muscle overuse due to hyperexcitability of trigeminal motoneurons.

Van Merkesteyn et al. have conducted extensive research on the non-surgical treatment of the non-suppurative Garre’s osteomyelitis of the jaws [19]. The management sequence uses occlusal splint therapy and physiotherapy to stabilize the muscles and the TMJ. Further clinical research supports these findings [20] that tendoperiostitis is precipitated by overworked muscles.

Other cited treatment options include antibiotics, anti-inflammatory drugs, muscle relaxants, antiresorptive medications (bisphosphonates), conservative therapy (occlusal splint, physiotherapy), hyperbaric oxygen treatment and surgery. The most promising results seem to originate from conservative treatment options, even if the studies overall have low quality and include a limited amount of patients [21].

The main objectives of the orthodontic treatment for this patient were to stabilize the TMJs, to reduce growth of the lower third of the face and to close the anterior open bite and obtain a class I molar and canine relationship, while preventing further invasive treatments.

After splint therapy and physiotherapy for the TMJs, the second phase of treatment consisted of orthopedic therapy aimed at directing the natural growth pattern of the patient and guiding tooth eruption.

Due to the thickening of the inferior border of the mandible in the osteomyelitis, all the initial cephalometric values were altered. Although there was a decreased articular angle with anterior rotation of the mandible (which would usually be associated with a short lower face third), due to the osteomyelitis, there was an increased lower third of the face with lip incompetence.

Usually, in these growing dolichocephalic patients, there is maxillary vertical excess, and orthopedic treatment involves lateral teeth intrusion and inhibition of the maxillary vertical growth with high-pull head gear appliances [22].

However, in this particular case, the anterior open bite was of a dental nature. The tongue thrusting habit, which is a known etiological factor for this pathology [23], probably played an important role in the development of the anterior open bite. In addition, exposure of frontal upper teeth at rest and during smiling and speech revealed that there was no maxillary vertical excess. Therefore, orthodontic and orthopedic treatment for this patient involved intrusion of the lateral teeth and inhibition of their eruption, as well as deprogramming of the tongue thrusting, in order to close the open bite.

The orthopedic appliances used in the second phase of treatment (the mandibular acrylic splint and transpalatal arch with acrylic button) ensured that the intrusive forces needed to close the bite and diminish growth of the lower third of the face were in the physiological range (masticatory and swallowing forces).

In conclusion, all orthodontic treatment objectives were met, without aggravating the osteomyelitis. Using the natural growth pattern of the patient, good esthetic and functional results were obtained using the smallest amount of treatment available with the least amount of biological force.

## Data Availability

No new data were created or analyzed in this study. Data sharing is not applicable to this article.
